# Identification of *Schistosoma mansoni *microRNAs

**DOI:** 10.1186/1471-2164-12-47

**Published:** 2011-01-19

**Authors:** Mariana C Simões, Jonathan Lee, Appolinaire Djikeng, Gustavo C Cerqueira, Adhemar Zerlotini, Rosiane A da Silva-Pereira, Andrew R Dalby, Philip LoVerde, Najib M El-Sayed, Guilherme Oliveira

**Affiliations:** 1 Graduate Program in Bioinformatics, Universidade Federal de Minas Gerais, Av. Antonio Carlos 6627, Belo Horizonte, MG, Brazil; 2 Department of Statistics, Peter Medawar Building, South Parks Road, Oxford, UK; 3 J Craig Venter Institute (JCVI), 9704 Medical Center Drive, Rockville, MD 20850, USA; 4 Department of Cell Biology and Molecular Genetics and Center for Bioinformatics and Computational Biology, University of Maryland College Park, MD 20742, USA; 5 Institute for Genome Sciences, University of Maryland School of Medicine, Baltimore, MD, USA; 6 CEBio, Instituto Nacional de Ciência e Tecnologia em Doenças Tropicais, Laboratory of Cellular and Molecular Parasitology, Centro de Pesquisas René Rachou, Fundação Oswaldo Cruz, Av. Augusto de Lima 1715, Belo Horizonte, 30190-002, Brazil; 7 University of Texas Health Science Center, 7703 Floyd Curl Dr. Mail Code 7760, San Antonio, Texas 78229-3900, USA; 8 Biosciences eastern and central Africa - International Livestock Research Institute (BecA-ILRI) Hub, P.O. Box 30709 Nairobi, Kenya

## Abstract

**Background:**

MicroRNAs (miRNAs) constitute a class of single-stranded RNAs which play a crucial role in regulating development and controlling gene expression by targeting mRNAs and triggering either translation repression or messenger RNA (mRNA) degradation. miRNAs are widespread in eukaryotes and to date over 14,000 miRNAs have been identified by computational and experimental approaches. Several miRNAs are highly conserved across species. In *Schistosoma*, the full set of miRNAs and their expression patterns during development remain poorly understood. Here we report on the development and implementation of a homology-based detection strategy to search for miRNA genes in *Schistosoma mansoni*. In addition, we report results on the experimental detection of miRNAs by means of cDNA cloning and sequencing of size-fractionated RNA samples.

**Results:**

Homology search using the high-throughput pipeline was performed with all known miRNAs in miRBase. A total of 6,211 mature miRNAs were used as reference sequences and 110 unique *S. mansoni *sequences were returned by BLASTn analysis. The existing mature miRNAs that produced these hits are reported, as well as the locations of the homologous sequences in the *S. mansoni *genome. All BLAST hits aligned with at least 95% of the miRNA sequence, resulting in alignment lengths of 19-24 nt. Following several filtering steps, 15 potential miRNA candidates were identified using this approach. By sequencing small RNA cDNA libraries from adult worm pairs, we identified 211 novel miRNA candidates in the *S. mansoni *genome. Northern blot analysis was used to detect the expression of the 30 most frequent sequenced miRNAs and to compare the expression level of these miRNAs between the lung stage schistosomula and adult worm stages. Expression of 11 novel miRNAs was confirmed by northern blot analysis and some presented a stage-regulated expression pattern. Three miRNAs previously identified from *S. japonicum *were also present in *S. mansoni*.

**Conclusion:**

Evidence for the presence of miRNAs in *S. mansoni *is presented. The number of miRNAs detected by homology-based computational methods in *S. mansoni *is limited due to the lack of close relatives in the miRNA repository. In spite of this, the computational approach described here can likely be applied to the identification of pre-miRNA hairpins in other organisms. Construction and analysis of a small RNA library led to the experimental identification of 14 novel miRNAs from *S. mansoni *through a combination of molecular cloning, DNA sequencing and expression studies. Our results significantly expand the set of known miRNAs in multicellular parasites and provide a basis for understanding the structural and functional evolution of miRNAs in these metazoan parasites.

## Background

Small non-coding RNAs are increasingly providing insights into important aspects of the biology of many organisms [1,2]. They include small interfering RNAs (siRNAs) and microRNAs (miRNAs), which are hallmarks of two important processes involved in RNA silencing [3,4]. RNA silencing is a general process in which small RNA molecules derived from precursor dsRNA molecules trigger sequence-specific repression of gene expression [4-6].

miRNAs comprise a family of non-coding RNAs with approximately 21-25 nucleotides that down-regulate gene expression at the post-transcriptional level. miRNAs are generated from endogenous hairpin structures in the nucleus and play an important role in controlling diverse cellular functions in eukaryotes, including cell differentiation, development, apoptosis, and genome integrity [7-9]. *In vivo *experiments indicate a crucial role in cell proliferation and cell death processes for some miRNAs, including *lin-4 *and *let-7 *in *C. elegans*; *bantam *and *mir-14 *in *Drosophila*; and *mir-23 *in humans [10].

The current understanding of miRNA biogenesis involves a series of coordinated processes. Briefly, primary transcripts of miRNAs are processed in the nucleus by Drosha, an RNase III-like enzyme into pre-miRNA, which are first exported into the cytoplasm by exportin-5 and then processed into miRNAs by Dicer, another type III RNase [11-13].

The primary method of identifying miRNA genes has been to isolate, reverse transcribe, clone, and sequence small RNA molecules [14-16]. In animals, discovery of miRNA genes, by using molecular cloning based methods has been supplemented by systematic computational approaches that identify evolutionarily conserved miRNA genes. Bioinformatics tools search for patterns of sequence and secondary structure conservation that are characteristic of metazoan miRNA hairpin precursors [17-19]. However, considerable filtering must be performed to elucidate likely miRNA candidates. The 5' end of miRNAs is reported to have a perfect base alignment of at least 7 consecutive nucleotides, which enables their identification [14]. The most sensitive of these methods indicate that miRNAs constitute nearly 1% of all predicted genes in nematodes, flies, and mammals [19-21]. However, computationally predicted miRNAs must be experimentally confirmed.

Although the first miRNA was identified in 1993, it was not until 2001 that the breadth of the miRNA gene class was recognized with cloning and sequencing of more than one hundred miRNAs from worms, humans, mice, and other species [22,23]. However, no large-scale identification of miRNAs has been carried out in *Schistosoma mansoni*.

*Schistosoma mansoni *is a human parasite that is responsible for the neglected tropical disease schistosomiasis. The parasite infects approximately 90 million people worldwide, causing morbidity and eventually death in Central and South America and Africa [24]. Although schistosomicidal drugs and other control measures exist, the development of new control strategies is necessary. In recent years, increasing attention has emerged over siRNAs as therapeutical agents [25]. The emergence of gene ablation technologies based on the RNAi phenomenon has opened up new experimental opportunities. Recently, several reports on the use of RNAi for the studies of schistosomes were published [26,27]. In this context, we attempted to identify potential miRNAs in *S. mansoni*. We use complementary experimental and computational approaches. We developed a homology-filtering approach used in a high-throughput pipeline in which all known miRNA genes were used as reference miRNAs. Fifteen potential miRNA candidates were discovered in *S. mansoni *using this analysis. The pipeline automated some of the manual steps, in particular a rule-based filtering approach for extracting the candidate pre-miRNA sequence, and it can also be applied to other genomes. By sequencing small-RNA cDNA libraries, we provide experimental evidence for 211 potential miRNAs candidates. The identification of new miRNA in the *S. mansoni *genome presents relevant information that is likely to be important for parasite development and sexual maturation.

## Results and Discussion

### Experimental identification of miRNAs

#### Cloning of short RNAs from S. mansoni adult worm pairs

An adult worm cDNA library of small RNAs was constructed using an established method based on a sequential ligation of oligonucleotide adapters to a size-fractionated sample of small RNAs [28]. Concatenated DNA fragments (each fragment from one putative miRNA) were cloned into a plasmid vector to generate a library. A total of 582 recombinant clones randomly selected from the library were sequenced. Twelve hundred sequences were analyzed and show to contain ~2-3 small RNA sequence in the same vector. Size distribution of the non-redundant miRNA set ranged from 17 to 25 nt, although the majority contained 20-24 nt, 21 nt being the most abundant. To identify the putative origin of the cloned sequences, a FASTA search was performed against GenBank http://www.ncbi.nlm.nih.gov and the *S. mansoni *genome (version 4.0) [29]. Sequences that had significant homology to breakdown products of abundant non-coding (nc) RNAs such as rRNA and tRNA were eliminated. A total of 584 ncRNAs were grouped into 211 clusters and were identified as possible miRNA candidates (see additional file 1, Table S1: clustering of 584 sequenced miRNAs). One hundred and sixty-one miRNAs were represented in the library by only one read and 50 were represented by clusters with up to 32 sequences. Since miRNAs are believed to occur at a frequency of approximately 0.5-1.5% of the total genes in the genome, the 13,200 genes predicted for *S. mansoni *should have generated between 66 and 198 miRNAs [30,31]. Thus, the number of miRNAs experimentally observed is in the expected range.

We further screened the candidate sequences against a database of known miRNAs, miRBase (http://microrna.sanger.ac.uk; release 13.0) to compare our candidate *S. mansoni *miRNAs to miRNAs from different species. Some miRNAs showed a high degree of conservation. Forty-two sequences had at least one match with mature miRNAs from different metazoan miRNA families, such as miR-832, miR-71, miR-297, and let-7 (Table 1). For example, sma-miR-36 perfectly matched miRNA family miR-87 from different species demonstrating miRNA conservation among more than 10 species. Previous studies in *C. elegans *showed that this miRNA family is expressed throughout development [20]. The yield of probable miRNA candidates was much lower for this analysis with *S. mansoni *than analyses of species that contain closer relatives in miRBase. The closest relative to *S. mansoni *in miRBase is *Schmidtea mediterranea*. These two organisms belong to the same phylum, a relatively broad classification. The sequences that did not match any of the known miRNAs (170 sequences) were considered to be putative members of novel families of schistosome miRNAs.

**Table 1:** Small *S. mansoni *RNAs with matches to know microRNAs present in the miRBase.

*S. mansoni *miRNAs	Number of Hits	Best Hit Name	miRNA family
sma-miR-7	4	gma-miR171b	miR-171
sma-miR-9	2	ptr-miR-1303	miR-1303
sma-miR-16	1	gga-miR-1465	miR-1465
sma-miR-17	10	dre-miR-739	miR-197
sma-miR-21	1	ebv-miR-BART2	miR-BART2
sma-miR-24	10	ptr-miR-451	miR-451
sma-miR-31	10	hsa-miR-513b	miR-513
sma-miR-32	1	ath-miR832-5p	miR-832
sma-miR-33	3	tca-miR-71	miR-71
sma-miR-36	10	tca-miR-87	miR-87
sma-miR-37	1	cbr-miR-240	miR-240
sma-miR-40	4	ptr-miR-432	miR-432
sma-miR-47	1	sme-let-7c	let-7
sma-miR-77	1	ptc-miR472b	miR472
sma-miR-78	1	ath-miR832-5p	miR-832
sma-miR-86	1	hsa-miR-1268	miR-1268
sma-miR-96	10	ptr-let-7b	let-7
sma-miR-103	1	kshv-miR-K12-3	miR-K12
sma-miR-105	8	dya-miR-289	miR-289
sma-miR-122	1	gga-miR-1465	miR-1465
sma-miR-129	8	dya-miR-289	miR-289
sma-miR-141	2	osa-miR166i	miR-166
sma-miR-149	1	gga-miR-1810	miR-1810
sma-miR-150	1	mmu-miR-297b-3p	miR-297
sma-miR-156	3	tni-miR-101b	miR-101
sma-miR-170	1	cel-miR-78	miR-78
sma-miR-182	10	dre-miR-739	miR-739
sma-miR-187	1	mmu-miR-297b-3p	miR-297
sma-miR-205	1	ppt-miR896	miR-896
sma-miR-209	1	mmu-miR-297b-3p	miR-297
sma-miR-210	1	gga-miR-1810	miR-1810
sma-miR-213	1	ath-miR832-5p	miR-832
sma-miR-216	1	kshv-miR-K12-3	miR-K12
sma-miR-226	1	mmu-miR-297b-3p	miR-297
sma-miR-234	1	gga-miR-1810	miR-1810
sma-miR-241	1	odi-miR-1500	miR-1500
sma-miR-247	4	ptr-miR-432	miR-432
sma-miR-262	1	sme-let-7c	let-7
sma-miR-276	1	mmu-miR-297b-3p	miR-297
sma-miR-278	1	gga-miR-1810	miR-1810
sma-miR-283	2	ptr-miR-1299	miR-1299
sma-miR-284	1	mmu-miR-297b-3p	miR-297

#### Expression analysis of miRNAs in S. mansoni

**Figure 1:**
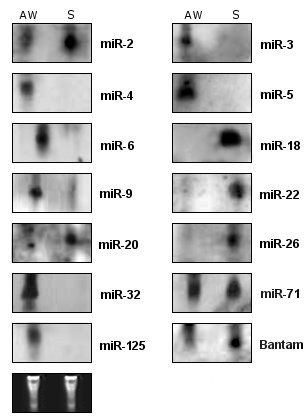
**Northern blot analysis of selected miRNAs in two different developmental stages in *S. mansoni***. Sixty micrograms of total RNA for each sample were separated on a 15% denaturing polyacrilamide gel, blotted and probed using miRNA specific DIG-labeled probes. Lanes from left to right: *S. mansoni *adult worm pairs (AW) and schistosomula (S) (7 days after mechanical transformation). miR-71, miR-125 and Bantam are the miRNAs identified in *S. mansoni *homolog to miRNAs of *S. japonicum *[38]. The tRNA and 5S rRNA bands were visualized by ethidium bromide staining of polyacrylamide gels and served as loading controls and are shown at the bottom.

The expression of miRNAs is tightly regulated in both time and space. Stage-specific or regulated miRNA expression suggests a role in development [20,21]. While high throughput techniques, such as microarray and next generation sequencing are being used, northern blot still remains the consensus method for validating miRNAs [32]. The frequency of reads of a specific miRNA in a non-normalized library can also be correlated with the expression level of that miRNA [33,34]. Based on that, the most abundant candidate miRNAs were further examined by northern blot to test for expression. Northern blots of total RNA from a mixture of male and female *S. mansoni *adult worms and the intramammalian larval stage, schistosomula, were hybridized with biotin-labeled probes. Previous studies analyzed the expression pattern of the Dicer gene during different life stages of *S. mansoni*. A threefold higher expression level was detected in seven day old schistosomula in comparison to the adult worm pairs [35]. It is possible that higher Dicer gene expression at this time was selected for the control of retrotransposon activation that may be more prone to occur during this period of active larval cell division and growth [36]. We detected the expression for 11 of 30 miRNAs in at least one of the 2 analyzed stages (Figure 1). We also analyzed in *S. mansoni *the expression of the five novel miRNAs recently identified in *S. japonicum *(sja-let-7, sja-miR-71, sja-bantam, sja-miR-125 and sja-miR-new1) [37]. Three (sja-miR-71 - non-specific, sja-bantam - schistosomula specific and sja-miR-125 - adult worm specific) of the five probes had a hybridization signal that was characteristic of miRNAs, demonstrating evolutionary conservation. Although the expression of sja-miR-71 and sja-bantam dropped quickly in *S. japonicum *lung-stage schistosomulum, we observed a strong hybridization signal for both miRNAs in *S. mansoni *(Figure 1) [37]. The other 2 candidates detected in *S. japonicum *(new-1 and let-7) may be expressed in other life cycle stages or in undetectable amounts in *S. mansoni *in the life cycle stages tested. We observed 2 miRNAs (mir-2 and mir-71) expressed in both life cycle stages tested, 7 in adult worms only (mir-4, mir-6, mir-9, mir-32, mir-125, mir-3, mir-5) and 5 in schistosomula only (mir-20, mir-18, mir-22, mir-26, Bantam). These results suggest a role for these miRNAs over the life cycle *stages *of *S. mansoni *possibly mediating important processes in the parasite growth and development.

Sequence analysis indicated miR-1 as the most abundant miRNA (32 reads). Although sequencing-based miRNAs expression profiling is a tool for measuring the relative abundance of miRNAs, the expression of the miR-1 was not detected by northern blot. In contrast, miR-32 is represented only by 2 clones in our sequences, which indicates a 12-fold lower expression level compared to that of miR-3 (24 reads). However, our small RNA blot analysis indicated that miR-32 was more abundantly expressed than miR-3 in the adult worm stage (Figure 1). The discrepancies between the cloning frequency and small RNA blot results could not be attributed to variations in RNA content because the same RNA samples were used for both experiments. One possible explanation could be bias in cloning efficiencies, or differential turnover rates of these miRNAs [38].

**Figure 2:**
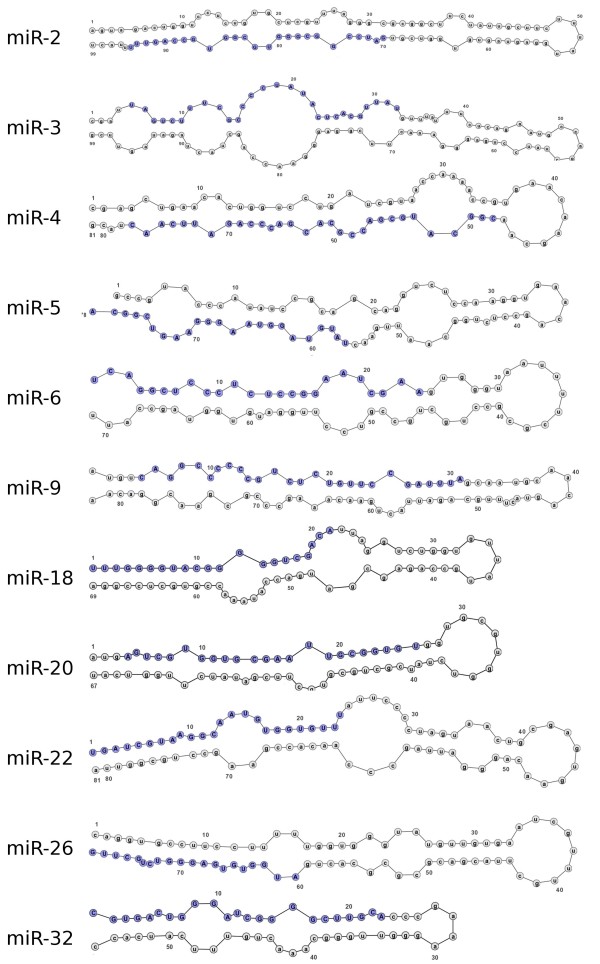
**Pre-miRNA secondary structure of selected novel *S. mansoni *miRNAs identified in small RNA libraries from adult worms and verified by northern blot analysis in adult worm and schistosomula stages**. *S. mansoni *genomic sequences upstream and downstream of the novel miRNAs analyzed with RNAfold from the Vienna RNA package. Dark blue text represents the mature miRNA sequence (20-25 nt).

The best method to differentiate miRNA from other endogenous small RNA is the ability of flanking sequences to adopt a pre-miRNA fold-back structure with the mature miRNA properly positioned within one of its strands enabling Dicer processing [17]. Eleven (36%) of the 30 potential miRNA detected by northern blot were mapped to ~500 different locations on the genome. To assess which of the regions corresponded to the real location of the possible miRNA gene, their secondary structures were studied using the Vienna RNAfold package http://rna.tbi.univie.ac.at/cgi-bin/RNAfold.cgi. Each image generated was visually inspected. A non-redundant set of 26 potential miRNA sequences were predicted to be capable of forming stem-loop structures characteristic of miRNA precursors, 11 of them were also confirmed by northern blot (Figure 2, see additional file 2, Figure S1: miRNA structures not confirmed by northern blot). Our results also show that multiple hairpin precursors for the same miRNA were observed in more than one location in the parasite genome (data not shown), pointing to the possibility that the same mature miRNA may be transcribed from more than one miRNA gene. Next, the miRNA genomic location was analyzed by BLAST against the *S. mansoni *genome. The selected miRNAs genes were observed to be located on intergenic regions, in agreement with published results [39-41].

### Computational identification of miRNAs

#### Homology search

The high-throughput homology search pipeline was performed with all known miRNAs in miRBase (release 13.0). In total, 6,211 mature miRNAs were used as reference sequences. The e-value cutoff for this analysis was set at 0.01. A total of 180 hits were registered. We observed 110 unique *S. mansoni *sequences, and 15 sequences were represented multiple times. For the BLASTn results see additional file 3, Table S2: high-throughput pipeline homology search results. The existing mature miRNAs that produced these hits are reported, as well as the locations of the homologous sequences in the *S. mansoni *genome. All hits aligned with at least 95% of the miRNA sequence, resulting in alignment lengths of 19-24 nt. All of the 110 unique mature miRNA candidates returned by the BLASTn search were assigned an analysis identifier with prefix 'SMan'.

#### RNA folding

The extended sequences (with additional 50 nt flanking the mature sequence) were folded with RNAshapes [42]. For the complete results for the extended sequence folding see additional file 4, Table S3: high-throughput pipeline extended sequence folding results.

Mean free energy (MFE) is a widely used criterion for filtering RNA folding results, and was observed to be an important filtering step in this analysis as well. The rationale for how MFE thresholds are derived, however, is not obvious when examining the literature. In fact, the guidelines proposed for uniform determination and annotation of miRNAs given by Ambros *et al. *do not mention MFE thresholds. Instead, the guidelines merely suggest that to be considered a miRNA, a candidate's lowest MFE fold should be a hairpin [17].

Determination of MFE threshold is dependent on tolerance to false positives, i.e. higher MFE thresholds result in inclusion of more candidates. A threshold of -20 kcal/mol has been generally used, but levels as high as -12 kcal/mol have also been explored [43]. We used a middle value of -15 kcal/mol as this genome has not been previously explored.

From the 110 unique *S*. *mansoni *sequences returned from the BLASTn search, 66 displayed MFE values of -15 kcal/mol or less when folded with RNAshapes. Forty three hairpins had MFE values greater than -15 kcal/mol.

All 66 of the extended sequences with MFE ≤ -15 kcal/mol displayed hairpins in at least one portion of the sequence when folded by RNAshapes. At ~122nt in length, the sequence is considerably longer than a typical miRNA hairpin, and as a result, only the ~70nt surrounding the mature sequence are of interest. This region was considered the candidate pre-miRNA sequence. In each of the 66 hairpins detected, the region surrounding the mature sequence was within a hairpin.

Of the 66 hairpins with MFE ≤ -15 kcal/mol, 36 structures contained the mature sequence entirely within the stem. The other 30 sequences contained the mature sequence partially or completely in a loop.

**Figure 3:**
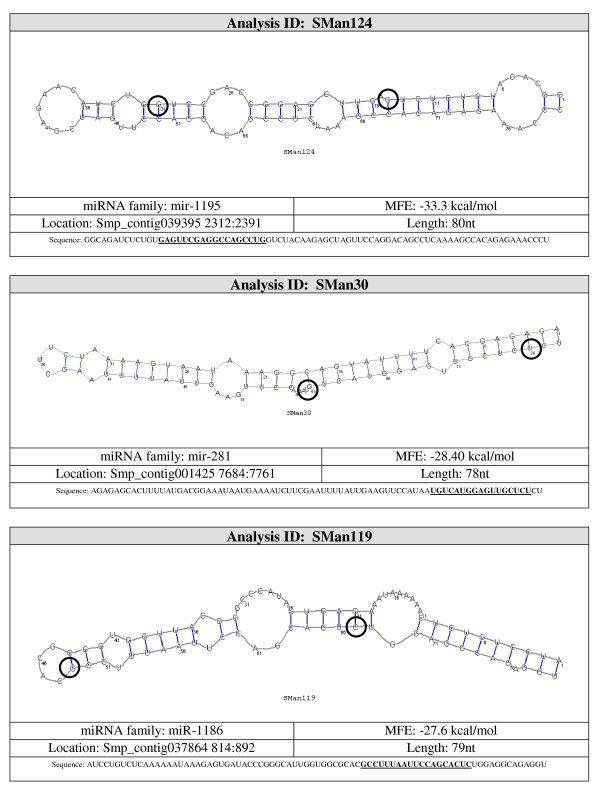
**Probable miRNA candidates SMan124, SMan30 and SMan119 identified by homology search**. For each structure, the location in the *S. mansoni *genome (version 4.0), pre-miRNA sequence, miRNA family, sequence length and MFE are given. The start and end of the mature sequence are circled in the structure. The mature sequence is also bolded and underlined in the pre-miRNA sequence.

**Figure 4:**
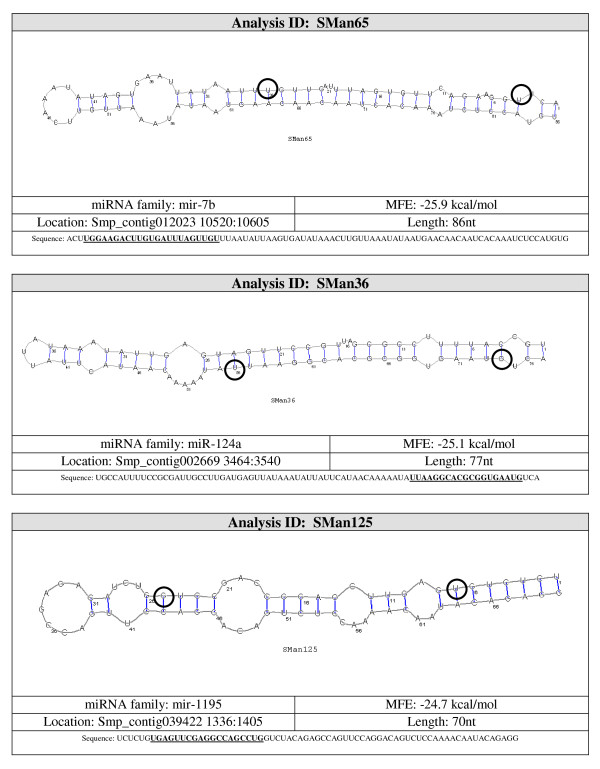
**Probable miRNA candidates SMan65, SMan36 and SMan125 identified by homology search**. For each structure, the location in the *S. mansoni *genome (version 4.0), pre-miRNA sequence, miRNA family, sequence length and MFE are given. The start and end of the mature sequence are circled in the structure. The mature sequence is also bolded and underlined in the pre-miRNA sequence.

**Figure 5:**
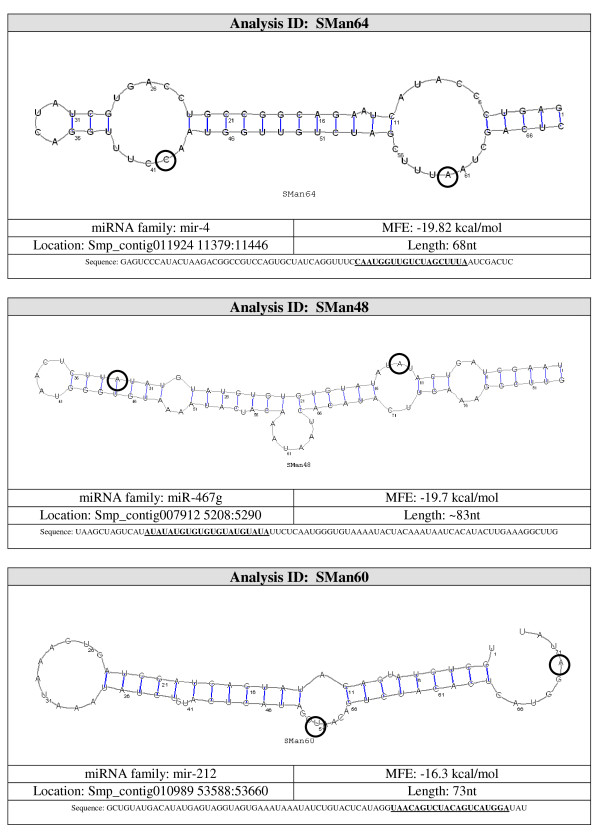
**Probable miRNA candidates SMan64, SMan48 and SMan60 identified by homology search**. For each structure, the location in the *S. mansoni *genome (version 4.0), pre-miRNA sequence, miRNA family, sequence length and MFE are given. The start and end of the mature sequence are circled in the structure. The mature sequence is also bolded and underlined in the pre-miRNA sequence.

**Figure 6:**
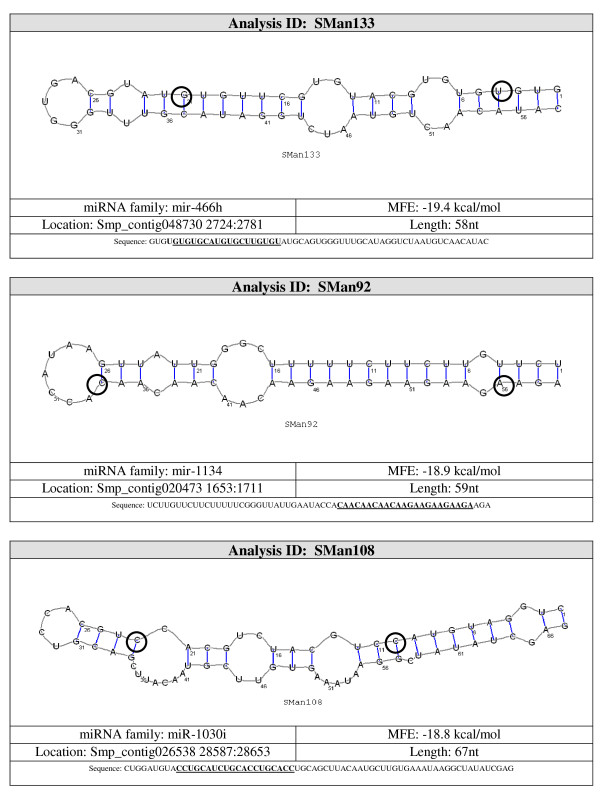
**Probable miRNA candidates SMan133, SMan92 and SMan108 identified by homology search**. For each structure, the location in the *S. mansoni *genome (version 4.0), pre-miRNA sequence, miRNA family, sequence length and MFE are given. The start and end of the mature sequence are circled in the structure. The mature sequence is also bolded and underlined in the pre-miRNA sequence.

**Figure 7:**
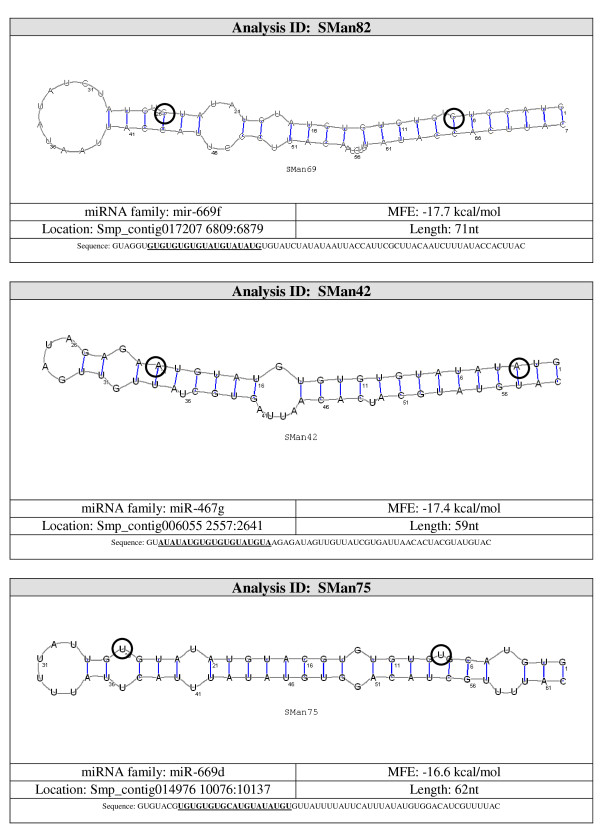
**Probable miRNA candidates SMan82, SMan42 and SMan75 identified by homology search**. For each structure, the location in the *S. mansoni *genome (version 4.0), pre-miRNA sequence, miRNA family, sequence length and MFE are given. The start and end of the mature sequence are circled in the structure. The mature sequence is also bolded and underlined in the pre-miRNA sequence.

Candidate pre-miRNA sequences were extracted from the 36 remaining hairpins and were refolded with RNAshapes. After refolding, 15 candidate pre-miRNAs had MFE ≤ -15 kcal/mol. These 15 sequences were considered to be likely pre-miRNA sequences. A summary of the results and the structures for the pre-miRNAs candidates are shown in Figures 3, 4, 5, 6 and 7.

The yield of probable miRNA candidates was much lower for this analysis with *S. mansoni *than analyses of species that contain closer relatives in miRBase. The findings suggest that it may have been difficult to find a large number of miRNAs in this analysis, due to the possibility of a large amount of sequence divergence between *S. mansoni *and its closest relative in miRBase is *S. mediterranea*. On the contrary, if one was interested in studying miRNAs in a mammal not found in miRBase, one would find 22 members from the same class and over 2,500 miRNA sequences in miRBase. However, for now, organisms such as *S. mansoni *must continue to have a mix of computational and experimental approaches, with an emphasis on experimental discovery.

#### Comparison of results to existing work

The high-throughput pipeline yielded fifteen probable pre-miRNA candidates. This number is comparable to the number of miRNAs found by Palokodeti *et al.*, who identified ten miRNA candidates in *S. mediterranea *by using all known human, *Drosophila *and *C. elegans *miRNAs as reference sequences [44]. However, the yields for the *S. mediterranea *analysis and for this analysis with *S. mansoni *are considerably smaller than other studies that have used homology methods with multiple genomes. Luo *et al. *identified 118 miRNAs in *Tribolium castaneum *(red flour beetle) using all available metazoan miRNAs as reference sequences [45]. Zhou *et al. *using a homology-based computational approach, found 300 human miRNA homologs in the domestic dog using only human miRNAs as the reference miRNAs [46]. Furthermore, Baev *et al. *identified 639 chimpanzee miRNAs with a homology-based approach, also using only human pre-miRNA sequences as a reference set [47]. Recently, novel miRNAs were identified in *Schistosoma japonicum*, a close relative of *S. mansoni *[37,48-50]. Chatterjee and Chaudhuri detected 489 homologous miRNA sequences in the *Anopheles gambiae *genome, using only *Drosophila *miRNA as the query sequence [51]. *Drosophila *is a well represented group of organisms in miRBase, but it is also in the same order as *A. gambiae*. As a result, this close relationship produced a large number of hits. Vertebrates, especially mammals, are currently the most represented organisms in miRBase, with respect to number of species and number of miRNA sequences.

It is worthwhile to note the extent to which the yield decreases as the distance between species grows. As a result, these findings suggest that the yield of a homology-based analysis is very dependent on the available content of miRBase. Artzi *et al. *used their recently released homology search web-server, miRNAminer, to increase the number of miRBase miRNAs for seven mammals by 50%, identifying 790 new miRNAs [52]. The strategy and filtering steps used by miRNAminer are very similar to those used in this paper, but moderately more comprehensive.

#### Analysis of homology search hits by species

**Figure 8:**
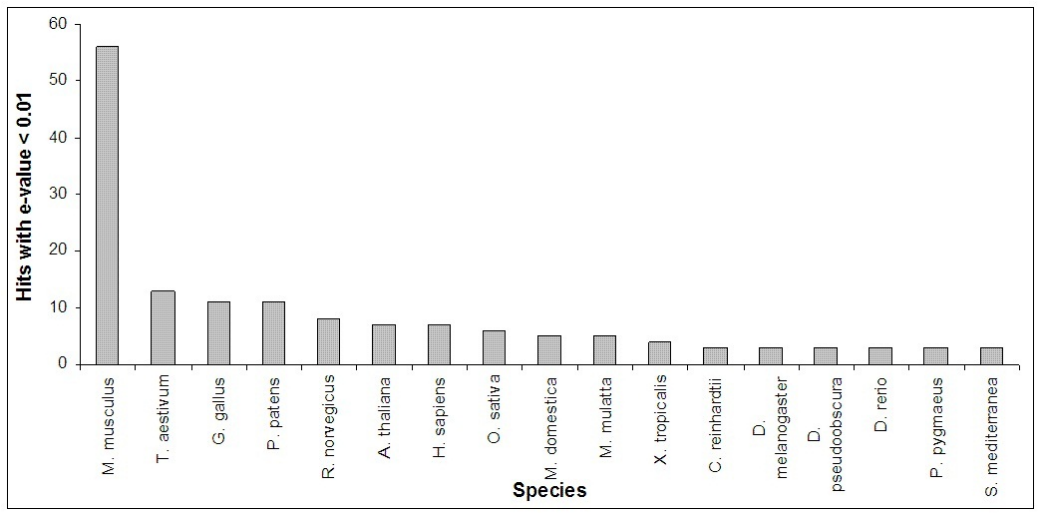
**Number of microRNAs hits per species with three or more hits with e-value < 0.01**. A homology search for the high-throughput pipeline was performed. Seventeen species had at least three hits with e-values < 0.01. An additional eighteen species had one or two hits.

As shown in Figure 8, when the homology search for the high-throughput pipeline was performed against all known miRNAs in miRBase, seventeen species had at least three or more hits with e-values < 0.01. An additional eleven species had two hits, and another seven species had one hit. *Mus musculus *displayed the highest number of hits with 56, over four times the number of hits for the next most represented species, *Triticum aestivum*. Although *M. musculus *has a high number (488) of miRNA sequences in miRBase, it does not appear that the sheer number of sequences is solely responsible for the higher frequency of hits. Other organisms are also well represented in miRBase, but displayed relatively few hits. For example, 695 human miRNAs are recorded in miRBase, but only five hits were observed in this study. Including the eight hits from *Rattus norvegicus*, over one-third of all hits observed were from the order, Rodentia. The cause of this observation is unclear. These findings may be merely the result of incomplete coverage of the database, which is only capturing a small number of the actual miRNAs present in mammals, or it may be possible that the miRNAs that have been identified in these Rodentia species are particularly well conserved across species.

**Figure 9:**
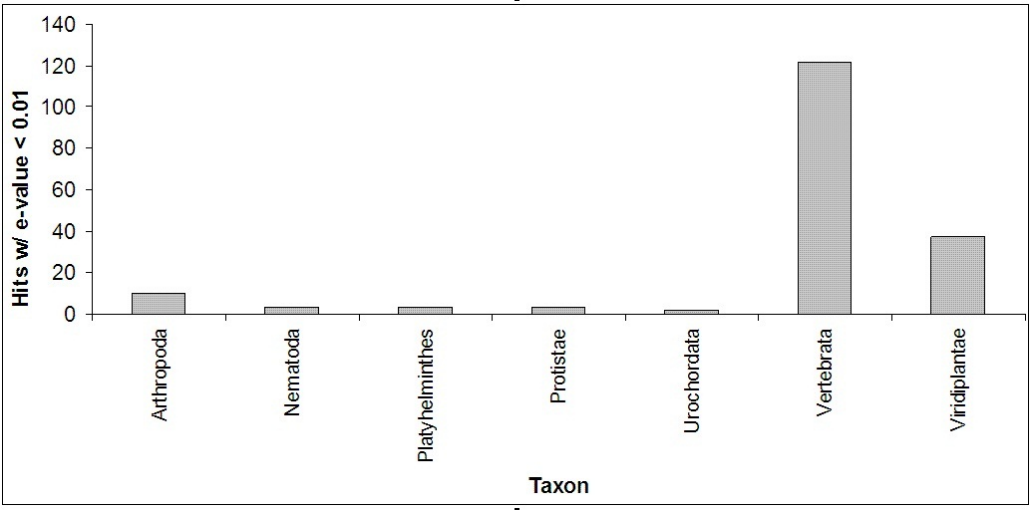
**Homology search hits by taxon. The number of hits with e-values < 0.01 for each major taxon listed in miRBase (subphylum, phylum or kingdom) is shown**.

Both metazoan and non-metazoan miRNAs were used as reference miRNAs in this analysis. It was assumed that metazoan miRNAs would be more likely to yield hits in *S. mansoni*, and that the non-metazoan sequences would provide somewhat of a negative control for the method, i.e. few hits should be observed with non-metazoan sequences. Interestingly, four of the top eight represented species are plants: *T. aestivum *(13 hits), *Physcomitrella patens *(11 hits), *Arabidopsis thaliana *(7 hits) and *Oryza sativa *(6 hits). The number of hits with e-values < 0.01 for each major taxon listed in miRBase (subphylum, phylum or kingdom) is shown in Figure 9. With 37 hits, plants (Viridiplantae) represent 20% of the total number of hits with e-values < 0.01. The most represented taxon is the subphylum, Vertebrata, with 122 hits or 68% of the total hits. This finding is not surprising as Vertebrata is also the taxon with the most number of miRNAs in miRBase (5157), more than three times the number of miRNAs in the next largest taxa, Viridiplantae (1638) and Arthropoda (1194).

**Figure 10:**
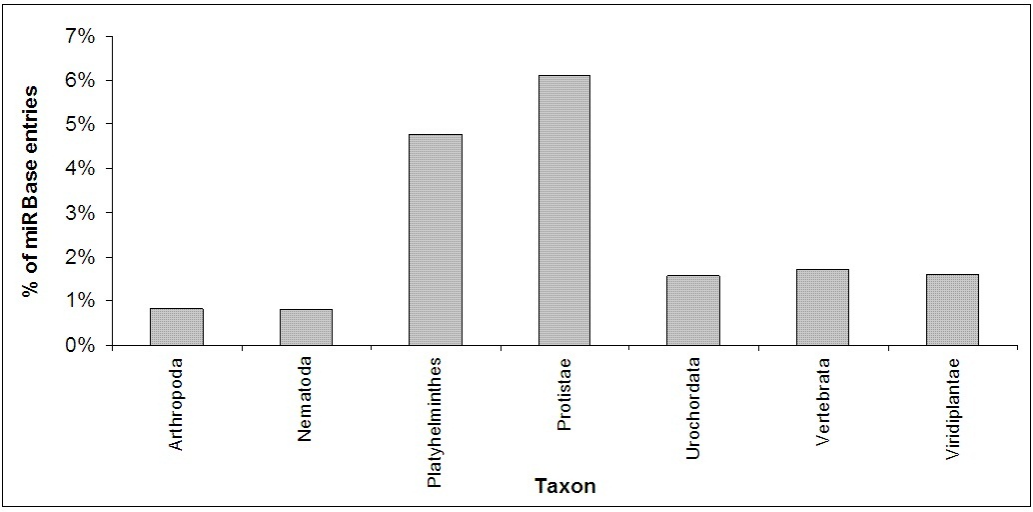
**Homology search hits as a percentage of miRBase entries**. The percentages of miRNAs from each major taxon in miRBase that returned a hit with e-value < 0.01 are shown.

The percentages of miRNAs from each major taxon in miRBase that returned a hit with e-value < 0.01 are shown in Figure 10. The kingdom Protistae (6.1%) and phylum Platyhelminthes (4.8%) display the highest percentages of hits. However, both of these taxa contain only one organism in miRBase, with each organism containing less than 65 miRNAs. Of the three taxa that have the most representatives in miRBase, Vertebrata and Viridiplantae display similar percentages (1.6-1.7%), and are both higher than Arthropoda (0.8%). It is interesting that a higher frequency of hits is observed for Viridiplantae than for Arthropoda, considering *S. mansoni *and Arthropoda would be more closely related as both are metazoans. This is a further indication that the number of miRNAs is likely to be much higher than that currently represented within the database.

#### Observed miRNA families

**Figure 11:**
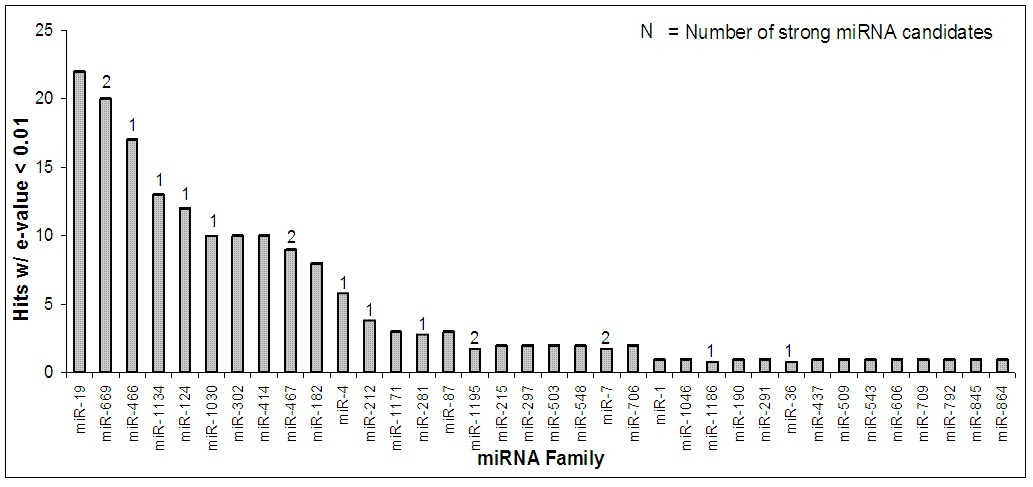
**Frequency of homology search hits by miRNA family**.

As shown in Figure 11, 36 different miRNA families were observed in the homology search. Of these, 22 families were observed multiple times, either from different species or within the same species. The miRNA family observed most frequently was miR-19, with 22 hits. Also shown in Figure 11 is the number of probable miRNA candidates that were observed in each family. Five of the six families with the most hits displayed at least one probable miRNA candidate. Ten of the thirteen families that displayed probable miRNA candidates rank in the top sixteen families with respect to number of hits. These results suggest that miRNA families that are highly conserved, appearing in the most number of species, may be most likely to yield probable miRNA candidates.

## Conclusions

The discovery of small regulatory RNA molecules, miRNAs, is undoubtedly one of the most important recent findings in biological research. This study demonstrates for the first time the presence of miRNAs in *S. mansoni *identified by complementary experimental and computational approaches. By cloning and sequencing of 1200 sequences from a small RNA library, 211 potential miRNA candidates were identified, of which 26 were predicted to form stem-loop structures characteristic of miRNA precursors. The expression of 14 of them was confirmed by northern blot analysis. The homology search by the high-throughput pipeline was performed with all known miRNAs in miRBase and fifteen novel likely miRNAs were detected in the parasitic organism *S. mansoni*. The identification of miRNA in the *S. mansoni *genome presents relevant information that is likely to be important to study various aspects as parasite development, gene regulation, evolutionary processes and sexual maturation.

## Methods

### Parasites and nucleic acid extraction

Total RNA was extracted from adult worm pairs and lung-stage schistosomula of *S. mansoni *with use of Trizol^® ^(Invitrogen). Cercariae were obtained from infected *Biomphalaria glabrata *snails and isolated parasite bodies were prepared as previously described [53]. Schistosomula were cultured for 7 days in complete RPMI medium supplemented with 10 mM Hepes, 2 mM glutamate, 5% fetal calf serum and antibiotics (100 U/ml penicillin and 100 μg/ml streptomycin) at 37°C in a 5% CO^2 ^atmosphere.

### RNA isolation and miRNA cloning

A total of 5 aliquots with 200 μg of total RNA isolated from adult worms by guanidine thiocyanate phenol-chloroform extraction were pooled [54]. The short RNA fraction ranging from 17 to 26 nt was purified and cloned as described in Chappell *et al. *[28]. Briefly, the concentration was quantified using the NanoDrop Spectrophotometer (NanoDrop Technologies, USA). Total RNA (1 mg) was resolved by electrophoresis on 15% denaturing polyacrylamide gel (8 M urea, 1 × TBE buffer), and short RNAs (17 to 26 nucleotides in length) were excised and eluted in 3 M NaCl solution at 4°C for 16 h. The gel purified small RNAs were dephosphorylated using APex™ Heat-Labile Alkaline Phosphatase (Epicentre) and ligated directly to a 5'-phosphorylated 3'-adapter oligonucleotide with a blocked 3'-hydroxyl terminus (5'-pUUUaaccgcatccttctcx-3'; uppercase, RNA; lowercase, DNA; p, phosphate; x, inverted deoxythymidine) (Dharmacon Research, Boulder, CO) to prevent self-ligation. The ligation products were separated from the excess of 3'-adapter on a 15% denaturing polyacrylamide gel and were subsequently ligated to a non-phosphorylated 5'-adater oligonucleotide (5'-tactaatacgactcactAAA-3'; uppercase, RNA; lowercase, DNA) (Dharmacon Research, Boulder, CO) using T4 RNA ligase (Invitrogen). The final products were again gel purified by size fractionation and submitted to reverse transcription reaction using the RT primer (5'-TTTT**CTGCAG**AAGGATGCGGTTAAA-3'; bold, *Pst*I site). This was followed by high fidelity PCR amplification using the reverse (RT primer) and forward (5'-AAA**CCATGG**TACTAATACGACTCACTAAA-3'; bold, *Nco*I site). The PCR products were digested with *Pst*I and *Nco*I and subsequently concatenated using T4 DNA ligase. The ends of the concatamers were filled in with Klenow/AT-tailing and ligated into a 2.1 TOPO TA vector (Invitrogen). Ligated plasmids were transformed into TOP10 cells (Invitrogen). The libraries were plated on Luria-Bertani (LB) ampicillin (100 μg/ml) plates and individual colonies were picked and put into 96-well plates containing LB ampicillin and grown overnight at 37°C with continuous shaking. The recombinant clones were selected, sequenced and the data was analyzed as described below.

### Computational analysis of microRNA library sequences

Base calling and quality trimming of sequence chromatograms were conducted using PHRED [55]. After masking of vector and adapter sequences using EMBOSS-restrict http://bioweb2.pasteur.fr/docs/EMBOSS/restrict.html, small RNA sequences ranging 17-25 nt in length were aligned by ClustalW2 program and redundant sequences removed [56].

The unique sequences were used in BLAST searches against the *S. mansoni *genome and miRBase database (http://microrna.sanger.ac.uk; release 13.0) to identify sequences from other species that closely match candidate *S. mansoni *miRNAs and removal of contaminating mRNAs, tRNAs, rRNAs, and other small RNAs. To predict the secondary structure of the remaining small RNA, Perl scripts were implemented to align the sequences to the genome of the parasite *S. mansoni *(http://www.schistodb.net Genome version 4.0) aiming at retrieving all possible genomic locations. In brief, the script executes BLAST to perform sequence similarity analysis and the result is parsed to retrieve the genomic positions to which each miRNA aligns [57]. In the next step, the script builds a FASTA file containing two sets of approximately 500 entries for each miRNA: one set of genomic sequences plus 40, 50, 60 or 70 nucleotides upstream and downstream. The secondary structures were predicted for each sequence using RNAfold from the Vienna RNA package [58]. Each image was further visually inspected to confirm the presence of a typical stem-loop conformation of pre-miRNAs. Among all structures created for each sequence, the one containing the mature miRNA in one arm of the hairpin precursor and with lowest folding free energy was selected. The final images were created using the VARNA http://varna.lri.fr/index.html to insert subtitles and highlight the mature miRNA sequence.

### miRNA expression analysis

For the northern blot analysis, total RNA from adult worm pairs and 7-day *in vitro *cultured schistosomula were used. Sixty micrograms of total RNA were separated on 15% denaturing polyacrylamide gels and electrotransferred to Hybond N+ membranes (GE Healthcare) in 1x Tris Borate EDTA using the Mini Trans-Blot Cell apparatus (Bio-Rad), according to the manufacturer's instructions. Membranes were UV cross-linked in the *UV Stratalinker*^® ^(Stratagene) and pre-hybridized in DIG Easy Hyb solution (Roche) at 37°C for 30 min. DNA oligonucleotides complementary to the miRNA sequences were labeled with DIG Oligo 3'-End Labeling Kit, Second generation (Roche). Hybridization was performed overnight at 37°C with 3' digoxigenin-labeled RNA probes at 4.5 pmol/μl. The membranes were washed using the DIG Wash (Roche) and blocked with Block Buffer Set (Roche). In brief, blots were incubated in blocking solution for 1 hour and then in antibody solution (anti-DIG, alkaline phosphatase conjugated antibody, 250 mU/ml) for 30 min, followed by washing twice in washing buffer. After equilibration in detection buffer, blots were incubated with chemiluminescent substrate CSPD (Roche). Membranes were exposed to X-ray film for 20 minutes and the films were digitized using a transmission scanner GS-800 Calibrated Densitometer (Bio-Rad).

### Computational identification of additional miRNAs

The first step was a BLASTn search, performed with all mature miRNA sequences downloaded from miRBase (release 13.0) against the *S. mansoni *genome (version 4.0). The expectation value cutoff for the pipeline development was set at 0.01. Similarly to the analysis of the microRNA libraries, the candidate miRNA sequences from the *S. mansoni *genome, plus 50nt on each side of the candidate mature miRNA sequence were selected using the MATLAB Bioinformatics toolbox. These extended sequences were then used for further analysis with the understanding that the sequence contained the candidate mature sequence, candidate hairpin, and extra nucleotides. Extended candidate miRNA sequences were folded using the standalone version of RNAshapes, which generates multiple folds for each sequence, ranking them by MFE. Each image was further visually inspected to confirm the presence of a typical stem-loop conformation of pre-miRNAs. Among all structures created for each sequence, the one containing the mature miRNA in one arm of the hairpin precursor and with lowest folding free energy was selected.

### Visual inspection of miRNA secondary structures

During method development, a mix of rules-based filters (e.g. MFE) and manual/visual inspection of the folded extended miRNA were used to determine probable pre-miRNA candidates. In the development of the pipeline, with an emphasis on automation, the following rules-based filters were developed and implemented:

Folded extended miRNA sequences with MFE greater than -15 kcal/mol were removed.

**Figure 12:**
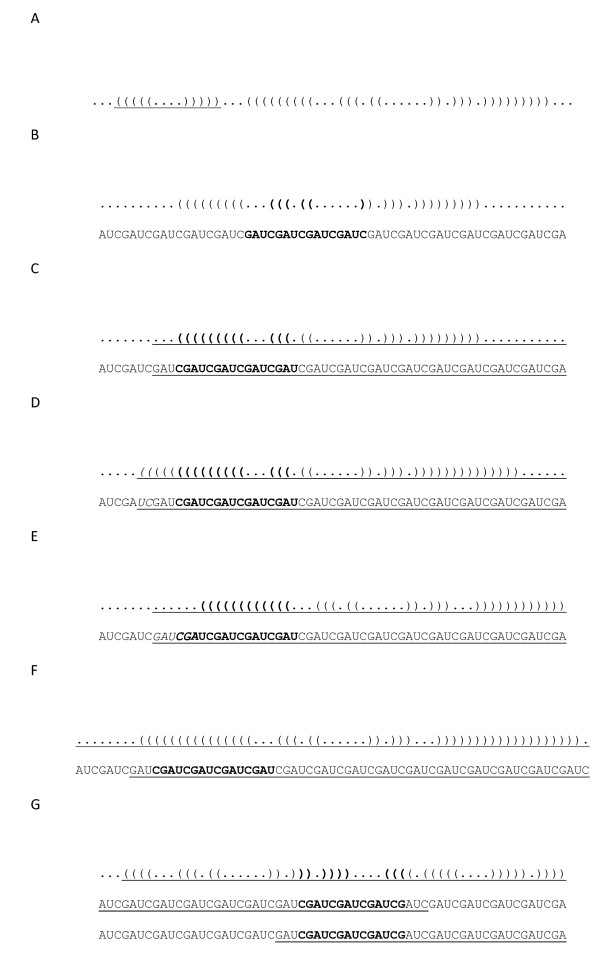
**Dot-bracket representation of miRNA folding**. Opposing sets of parentheses represent individual hairpins. Each parenthesis represents a paired base within a hairpin. Dots represent unpaired bases.

An example of the dot-bracket output is shown in Figure 12A. Opposing sets of parentheses represent individual hairpins. Each parenthesis represents a paired base within a hairpin. Dots represent unpaired bases. In the dot-bracket output in Figure 12A, the underlined portion represents one hairpin, with five paired bases on either stem and four bases in the loop. The entire dot-bracket output represents two hairpins with three unpaired bases on either end of the sequence.

Structures with the mature or partial miRNA contained in the loop of the hairpin were excluded, i.e. no bases in the mature miRNA can be represented by dots that are between opposing parentheses. For example, the structure in Figure 12B was filtered out of the analysis. The mature miRNA sequence is underlined in the sequence and in the dot-bracket diagram. If only one major hairpin is present, the candidate pre-miRNA sequence is found within the bases from 1) the third base from the end of the mature miRNA sequence away from the loop to 2) the last base on the hairpin end of the extended miRNA sequence. A major hairpin was defined as one that extends >75% of the extended miRNA sequence. The selected candidate pre-miRNA sequence is shown underlined in Figure 12C.

When selecting the candidate pre-miRNA, if additional paired bases were directly adjacent to the selected sequence, the selection was extended to include these bases. This step prevents known stems of the hairpin from being truncated. The two additional bases, 'UC' (shown italicized), on the left of the selected sequence in Figure 12D illustrates this rule.

If either end of the selected hairpin sequence terminates in paired bases, while the other end of the sequence terminates in unpaired bases, the paired end of the sequence was extended by the number of unpaired bases on the other end. Extending the sequence required extracting the additional bases from the *S. mansoni *database. The rationale for this rule was that unpaired bases on the miRNA end may not actually be unpaired, but instead the bases that they pair with were merely not present in the original extended sequence. After the additional bases were added, the sequence was refolded. If the newly added bases were unpaired, the original fold was used. In the example in Figure 12E, the six bases on the left of the selected sequence, 'GAUCGA', were unpaired. However, the other end of the selected sequence ended in paired bases. As a result, six additional bases were added and the sequence was refolded as shown in Figure 12F.

In cases where two or more hairpins were present in the extended sequence, two sequence selections are made, i.e. on either side of the mature sequence. The rules described above were then followed as shown in Figure 12G.

Unpaired bases at the ends of the hairpin stems that are not part of the mature miRNA sequence or the 3nt extension were removed.

The candidate pre-miRNA sequences were folded using RNAshapes. Structures with MFE ≤ -15 kcal/mol were considered probable pre-miRNA sequences.

## Authors' contributions

MCS performed all experiments and wrote the paper. AD directed the miRNA library construction and MCS, GCC and AZ carried out the prediction and computational analysis of miRNA libraries. JL and ARD performed the bioinformatics experiments. RASP contributed to the northern blot experiments. PLV, GO and NMES designed and directed the project. All authors read and approved the final manuscript.

## Supplementary Material

Additional file 1 **Table S1. Clustering of 584 sequenced miRNAs**. 584 sequenced miRNAs were grouped into 211 clusters. The putative miRNA ID, sequence, length, the number of sequences (Frequency) and genomic locations are shown. The results in Northern blot analysis are shown (+ positive signal, - neative signal, NT not tested). Known genomic locations are hyperlinked to http://www.schistodb.net. This Table is also available at http://www.cebio.org/content/2009/04/08/schistosoma-mansoni-micrornas. Click here for file

Additional file 2 **Figure S1. Predicted precursor structures of new *S. mansoni *miRNAs**. The miRNAs shown were undetected by northern blot in adult worm and schistosomula stages. The RNA secondary structure of the precursors was predicted using using RNAfold from the Vienna RNA package http://rna.tbi.univie.ac.at/cgi-bin/RNAfold.cgi. The file is also available at http://www.cebio.org/content/2009/04/08/schistosoma-mansoni-micrornas. Click here for file

Additional file 3 **Table S2. High-throughput pipeline homology search results**. Results from BLASTn homology search using all known mature miRNA sequences to search the *S. mansoni *miRNA database (e-value < 0.01). The file is available at http://www.cebio.org/content/2009/04/08/schistosoma-mansoni-micrornas. Click here for file

Additional file 4 **Table S3. High-throughput pipeline extended sequence folding results**. Results from RNAShapes folding of 110 extended miRNA sequences. The file is available at http://www.cebio.org/content/2009/04/08/schistosoma-mansoni-micrornas. Click here for file
