# Supplementary figures and images for: Identification of Schistosoma mansoni microRNAs
Source: BMC Genomics. 2011 Jan 19;12:47. doi: 10.1186/1471-2164-12-47 (PMC3034697; doi:10.1186/1471-2164-12-47)

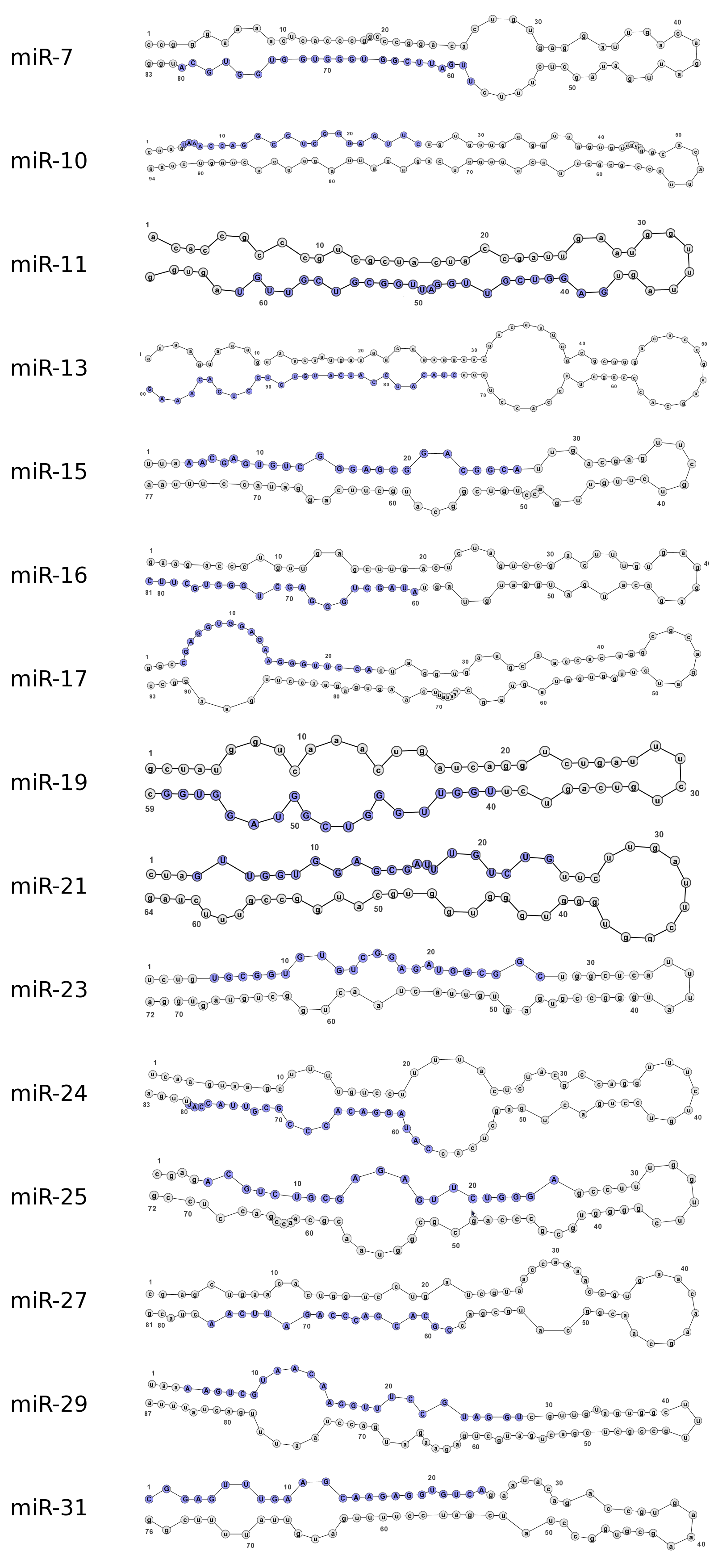

Supplement: Additional file 2 — Figure S1. Predicted precursor structures of new S. mansoni miRNAs. The miRNAs shown were undetected by northern blot in adult worm and schistosomula stages. The RNA secondary structure of the precursors was predicted using using RNAfold from the Vienna RNA package http://rna.tbi.univie.ac.at/cgi-bin/RNAfold.cgi. The file is also available at http://www.cebio.org/content/2009/04/08/schistosoma-mansoni-micrornas. [file 1471-2164-12-47-S2.PNG]
